# News and Events of the Week

**Published:** 1911-03-04

**Authors:** 


### News and Events of the Week.

At the annual general meeting of the Newsvendors' Benevolent and Provident Institution Charles Skeats was elected to be the recipient of "The Hospital" Pension for the year 1911.

Some interesting letters from Miss Florence Nightingale on health visitors for rural districts have been reprinted in a report originally issued in 1892 (to be obtained from the National League for Physical Education and Improvement, 4 Tavistock Square, W.C., or P. S. King and Son, Westminster, price 3d. net, postage 1d.). These letters show that the "Lady of the Lamp" was even then keenly alive to the necessity for health teaching in rural, no less than in urban, districts. Unfortunately, as is pointed out in the Preface, the cure of disease is far more popular, in spite of the greater cost, than its prevention, and as Miss Nightingale herself says, "there are more people to pick us up when we fall than to enable us to stand upon our feet." It is in the hope of stimulating the spread of health visiting that this pamphlet, which contains much that is suggestive and useful, in addition to the letters, has now been reprinted.

Dr. Infroit, the distinguished director of the laboratory of radiography at the Saint-Louis Hospital, Paris, has burned his hand so badly with radium that he has been obliged to have one of his fingers amputated.

A new journal entitled *Le Répertoire de Médecine Internationale* has just made its appearance in Paris. It has been founded with the object of giving the pith of the principal medical publications of the whole world in pamphlet form. It can be obtained from the Medical Library of Jules Rousset, 1 Rue Casimir-Delavigne, Paris, at a cost of 14 francs per annum.

His Excellency the Earl of Aberdeen, K.T., has consented to act as Patron of the twenty-sixth annual congress of the Royal Sanitary Institute, to be held at Belfast from July 24 to July 29, 1911. His Majesty the King is Patron of the institute. The Right Hon. Lord Dunleath, D.L., J.P., has consented to act as president of the Congress. A public meeting to inaugurate arrangements for the meeting was held at the City Hall, Belfast, on Tuesday, January 31. Communications relative to the institute and the Congress should be addressed to E. White Wallis, F.S.S., Secretary and Director, the Royal Sanitary Institute, 90 Buckingham Palace Road, London, S.W.

The ordinary general meeting of the Röntgen Society was held on Thursday, March 2, at 66 Victoria Street, Westminster, S.W., when a paper was read by A. A. Campbell Swinton on "Some Experiments with a 10,000-volt Storage Battery.'

The latest return of the Metropolitan Asylums Board (issued last night) showed that 557 cases of measles were under treatment in the Board's hospitals at midnight on Monday. The admissions during that day—thirty-four—comprised ten patients from Wandsworth, five from Poplar, three each from Islington and Lambeth, and two each from Hammersmith, St. Marylebone, Hackney, Bethnal Green, and Camberwell, and one each from Paddington, Holborn, and Bermondsey.

The following lectures at the Central London Throat and Ear Hospital, Gray's Inn Road, W.C., will be given this week: Tuesday, March 7, at 3.45 p.m., Pharynx and Naso-Pharynx, Mr. J. Gay French; Friday, March 10, at 3.45 p.m., Pharynx and Naso-Pharynx, Dr. Abercrombie.

At the thirteenth ordinary meeting of the Royal Society of Arts, John Street, Adelphi, London, W.C., on Wednesday evening, March 8, a paper on "Plague and its Dissemination" will be read by James Cantlie, M.A., M.B., C.M., F.R.C.S., D.P.H. The chair will be taken at eight o'clock by Sir Shirley Forster Murphy, F.R.C.S.

Two more cases of smallpox have been admitted to the Joyce Green Hospital from the Mile End Infirmary, and one from Bethnal Green, making the second notification from this parish since the outbreak. A "suspected" case from Leyton was received at the South Wharf Shelters during the day, but was subsequently "discharged" as not being one of smallpox. According to the official "return" thirty-eight patients were under treatment at midnight on Monday at the Joyce Green Hospital, and two at the South Wharf Shelters, Rotherhithe. Monday's admissions—to midnight—five in all, comprised one from Greenwich, two from Mile End, and two from Hackney. Three cases so far have been notified from this latter district. On Monday two deaths occurred. One was that of a female at the Rotherhithe Shelters, and the other also that of a female at the Joyce Green Hospital. There are now forty-three patients under treatment.

On Monday last Dr. Forbes Winslow lectured before the Psycho-Therapeutic Society at the Caxton Hall, Westminster on "The Power of Psycho-Therapeutics in dealing with Degeneration." He drew attention to the fact that degeneration had increased very much of late, and said that, in regard to it, psycho-therapeutical treatment, as a means of preventing its progress, had become of public importance especially of late in consequence of the report issued by the Home Office dealing with the emotional side of the question. This report drew attention to the fact that crime had very much increased during the last decade, which was largely due to the "general relaxation in public sentiment with regard to crime." It was shown by the report that the majority of the criminal class consisted chiefly of youths, who from weakness of character yielded to temptation. Dr. Winslow maintained that the power of suggestion judiciously used in the case of these youths would have the effect of, in many instances, preventing the spread of degeneration. This treatment would not, however, be of any avail in the use of the confirmed and hardened criminal. Dr. Winslow proceeded to analyse the reasons for this increase of degeneration and to consider how to deal with it psychically. The streets of London were full of degenerates, of one form or another, and 149,000 mentally defective persons were at the present time uncertified, unprotected, at large, and uncontrolled. Many of these were fit and proper subjects for judicious psycho-therapeutical treatment. At present many of them ultimately become either criminals or drunkards. Vice could be eradicated by the proper use of suggestion, so could the drink and drug habits. The wretched army of degenerates now at largo propagated their species and bred specimens like themselves. The number of pauper lunatics at present certified was 118,901.

The following are the arrangements for next week at the Medical Graduates' College and Polyclinic, 22 Chenies Street, W.C. : Monday, March 6, at 4 p.m., Dr. S. E. Dore, Clinique (Skin); at 5.15 p.m., Dr. George Pernet, "Pruritus." Tuesday, at 4 p.m., Dr. G. A. Sutherland, Clinique (Med.); at 5.15 p.m., Dr. Anton Lieven (Aix-la-Chapelle), Treatment of Syphilis bearing on the Results obtained by Ehrlich's '606.' Wednesday, at 4 p.m., Mr. Lenthal Cheatle, Clinique (Surg.); at 5.15 p.m., Dr. Essex Wynter, "Heart Disease in Middle Age." Thursday, at 4 p.m., Dr. A. Morison, Clinique (Med.); at 5.15 p.m., Dr. Herbert S. French, "The Vasomotor Factor in the Causation and Treatment of High Blood Pressure." Friday, at 4 p.m., Dr. St. Clair Thomson, Clinique (Ear, Nose and Throat).

The following are the arrangements for next week at the West London Postgraduate College, West London Hospital, Hammersmith Road : March 6 and 9, at 10 a.m., demonstration by Surgical Registrar; March 6, at 12 noon, pathological demonstration; March 7, at 11.30 a.m., demonstration of minor operations; March 8, at 10 a.m., gynæcological demonstration; March 9, at 12.15 p.m., lecture, Dr. Grainger Stewart, "Practical Medicine." Lectures at 5 p.m. : March 6, Mr. Dunn, "Some Common Forms of Eye Disease and their Treatment"; March 7, Dr. Robert Jones, "Heredity and its Physical Bases"; March 8, Dr. Beddard, "Practical Medicine" (lecture 6); March 9, Dr. Drummond Robinson, gynæcological or obstetrical section; March 10, Mr. Pardoe, "The Treatment of Acute and Chronic Urethritis."

